# Supplementary material for: A fault-intrusion-tolerant system and deadline-aware algorithm for scheduling scientific workflow in the cloud
Source: PeerJ Comput Sci. 2021 Nov 2;7:e747. doi: 10.7717/peerj-cs.747 (PMC8576554; doi:10.7717/peerj-cs.747)
Supplement: Supplemental Information 6 [file peerj-cs-07-747-s006.zip › Myproject1.1/docs/org/cloudbus/cloudsim/network/datacenter/NetworkConstants.html]

NetworkConstants


JavaScript is disabled on your browser.


- Overview
- Package
- Class
- Use
- Tree
- Deprecated
- Index
- Help

- Prev Class
- Next Class

- Frames
- No Frames

- All Classes

- Summary:
- Nested |
- Field |
- Constr |
- Method

- Detail:
- Field |
- Constr |
- Method


org.cloudbus.cloudsim.network.datacenter

## Class NetworkConstants

- java.lang.Object
- - org.cloudbus.cloudsim.network.datacenter.NetworkConstants

- ---

    

  ```
  public class NetworkConstants
  extends java.lang.Object
  ```

- - ### Field Summary

    Fields

    | Modifier and Type | Field and Description |
    | --- | --- |
    | `static int` | `Agg_LEVEL` |
    | `static double` | `AggSwitchPort` |
    | `static long` | `BandWidthAggRoot` |
    | `static long` | `BandWidthEdgeAgg` |
    | `static long` | `BandWidthEdgeHost` |
    | `static boolean` | `BASE` |
    | `static int` | `COMMUNICATION_LENGTH` |
    | `static int` | `currentAppId` |
    | `static int` | `currentCloudletId` |
    | `static int` | `EDGE_LEVEL` |
    | `static double` | `EdgeSwitchPort` |
    | `static int` | `EXECUTION` |
    | `static int` | `FILE_SIZE` |
    | `static int` | `FINISH` |
    | `static int` | `HOST_PEs` |
    | `static int` | `iteration` |
    | `static boolean` | `logflag` |
    | `static int` | `maxhostVM` |
    | `static double` | `maxMemperVM` |
    | `static int` | `nexttime` |
    | `static int` | `OUTPUT_SIZE` |
    | `static int` | `PES_NUMBER` |
    | `static int` | `ROOT_LEVEL` |
    | `static double` | `RootSwitchPort` |
    | `static double` | `seed` |
    | `static double` | `SwitchingDelayAgg` |
    | `static double` | `SwitchingDelayEdge` |
    | `static double` | `SwitchingDelayRoot` |
    | `static int` | `totaldatatransfer` |
    | `static int` | `WAIT_RECV` |
    | `static int` | `WAIT_SEND` |
  - ### Constructor Summary

    Constructors

    Constructor and Description

    `NetworkConstants()`
  - ### Method Summary

    - ### Methods inherited from class java.lang.Object

      `clone, equals, finalize, getClass, hashCode, notify, notifyAll, toString, wait, wait, wait`

- - ### Field Detail


    - #### maxhostVM

      ```
      public static int maxhostVM
      ```


    - #### HOST\_PEs

      ```
      public static int HOST_PEs
      ```


    - #### maxMemperVM

      ```
      public static double maxMemperVM
      ```


    - #### currentCloudletId

      ```
      public static int currentCloudletId
      ```


    - #### currentAppId

      ```
      public static int currentAppId
      ```


    - #### EXECUTION

      ```
      public static final int EXECUTION
      ```

      See Also:
      :   Constant Field Values


    - #### WAIT\_SEND

      ```
      public static final int WAIT_SEND
      ```

      See Also:
      :   Constant Field Values


    - #### WAIT\_RECV

      ```
      public static final int WAIT_RECV
      ```

      See Also:
      :   Constant Field Values


    - #### FINISH

      ```
      public static final int FINISH
      ```

      See Also:
      :   Constant Field Values


    - #### ROOT\_LEVEL

      ```
      public static final int ROOT_LEVEL
      ```

      See Also:
      :   Constant Field Values


    - #### Agg\_LEVEL

      ```
      public static final int Agg_LEVEL
      ```

      See Also:
      :   Constant Field Values


    - #### EDGE\_LEVEL

      ```
      public static final int EDGE_LEVEL
      ```

      See Also:
      :   Constant Field Values


    - #### PES\_NUMBER

      ```
      public static final int PES_NUMBER
      ```

      See Also:
      :   Constant Field Values


    - #### FILE\_SIZE

      ```
      public static final int FILE_SIZE
      ```

      See Also:
      :   Constant Field Values


    - #### OUTPUT\_SIZE

      ```
      public static final int OUTPUT_SIZE
      ```

      See Also:
      :   Constant Field Values


    - #### COMMUNICATION\_LENGTH

      ```
      public static final int COMMUNICATION_LENGTH
      ```

      See Also:
      :   Constant Field Values


    - #### BASE

      ```
      public static boolean BASE
      ```


    - #### BandWidthEdgeAgg

      ```
      public static long BandWidthEdgeAgg
      ```


    - #### BandWidthEdgeHost

      ```
      public static long BandWidthEdgeHost
      ```


    - #### BandWidthAggRoot

      ```
      public static long BandWidthAggRoot
      ```


    - #### SwitchingDelayRoot

      ```
      public static double SwitchingDelayRoot
      ```


    - #### SwitchingDelayAgg

      ```
      public static double SwitchingDelayAgg
      ```


    - #### SwitchingDelayEdge

      ```
      public static double SwitchingDelayEdge
      ```


    - #### EdgeSwitchPort

      ```
      public static double EdgeSwitchPort
      ```


    - #### AggSwitchPort

      ```
      public static double AggSwitchPort
      ```


    - #### RootSwitchPort

      ```
      public static double RootSwitchPort
      ```


    - #### seed

      ```
      public static double seed
      ```


    - #### logflag

      ```
      public static boolean logflag
      ```


    - #### iteration

      ```
      public static int iteration
      ```


    - #### nexttime

      ```
      public static int nexttime
      ```


    - #### totaldatatransfer

      ```
      public static int totaldatatransfer
      ```
  - ### Constructor Detail


    - #### NetworkConstants

      ```
      public NetworkConstants()
      ```


- Overview
- Package
- Class
- Use
- Tree
- Deprecated
- Index
- Help

- Prev Class
- Next Class

- Frames
- No Frames

- All Classes

- Summary:
- Nested |
- Field |
- Constr |
- Method

- Detail:
- Field |
- Constr |
- Method
